# Piceatannol and its analogues alleviate *Staphylococcus aureus* pathogenesis by targeting β-lactamase biofilms and α-hemolysin

**DOI:** 10.1038/s41598-025-89654-1

**Published:** 2025-02-14

**Authors:** Guizhen Wang, Jingyao Wen, Zizeng Tian, Hanbing Zhou, Xinli Peng, Peigang Zhang, Zhandong Li

**Affiliations:** 1 College of Biological and Food Engineering, Jilin Engineering Normal University, Changchun, 130052 China; 2 Jilin Engineering Normal University, Changchun, 130052 China

**Keywords:** *Staphylococcus aureus*, Biofilm, β-lactamase, α-hemolysin, Anti-infection, Antimicrobials, Bacteriology, Biofilms

## Abstract

β-Lactamases, biofilms and toxins pose challenges for combating *S. aureus* infection. Thus, identifying inhibitors that can restore bacterial sensitivity to antibiotics, destroy biofilms, and antitoxins is a promising way to develop alternative agents. In this study, we found that piceatannol (pit), along with its analogues resveratrol (ret) and pterostilbene (pts) bind with β-lactamase to inhibit its activity, and 96TYR, 58ILE and 66LYS were identified as the critical binding residues. Pit and pts reduced the ampicillin (Amp) and gentamicin (Gm) MICs against *S. aureus* and enhanced the bactericidal ability of Amp. Pit and its analogues inhibited the formation of *S. aureus* USA300. In addition, the pit analogues bound with α-hemolysin and suppressed the hemolysis activity of the bacterial culture supernatant. The mechanism analysis revealed that pit exhibited multiple potential binding modes with α-hemolysin. Pit significantly decreased the cytotoxicity and the adherence effect mediated by *S. aureus* and increased the survival rate of *Galleria mellonella* that infected with *S. aureus*, the pathological tissue damage of *Galleria mellonella* was alleviated by treatment with pit alone or in combination with Amp. Taken together, our findings identify promising compounds for the development of *S. aureus* infection inhibitors.

**Supplementary Information:**

The online version contains supplementary material available at 10.1038/s41598-025-89654-1.

## Introduction

*Staphylococcus aureus* (*S. aureus*) is the causative agent of pneumonia and other respiratory tract infections, as well as hospital-acquired bacteraemia^1^. The annual incidence of *S. aureus* bacteraemia is 20 to 50 cases per 100,000 people, with a mortality rate of 10–30% among infected patients^2,3^, which results in a significant public health burden. The increase in the number of *S. aureus* strains with antibiotic resistance, particularly the highly virulent methicillin-resistant *S. aureus* (MRSA) strains^4,5^, has led to increased rates of infection, mortality, and hospitalization, posing challenges in combating infections caused by this pathogen^6,7^.

The emergence of β-lactamases has hindered the efficacy of β-lactam antibiotics in the treatment of bacterial infections^8,9^. In addition, *S. aureus* is one of the most common pathogens that causes biofilm-associated clinical infections^10,11^, which can result in persistent infections due to the generation of persisters^12,13^, cause chronic disease and trigger serious complications, which prolong the treatment cycle and increase the difficulty of treatment. The ability of *S. aureus* to form biofilms has led to it becoming the most common pathogen in health care and community settings^14,15^. More than 80% of bacterial infections are accompanied by biofilm formation, with approximately 17 million new biofilm-associated infections occurring in the United States each year^16^. *S. aureus* α-hemolysin (Hla) is one of the most important exotoxins related to its pathogenicity. It can induce cell apoptosis by activating the MAPK signalling pathway^17–19^. Hla has also been shown to mediate inflammation and cell death by activating caspase-1, resulting in the release of IL-1β, IL-6 and IL-8^20,21^. Therefore, agents that target these critical systems to combat *S. aureus* infection have application potential.

Piceatannol (pit) and its analogues resveratrol (ret) and pterostilbene (pts) are bioactivators that are sourced mainly from food materials, including but not limited to blueberries, grapes, and peanuts; these analogues have various biological activities, such as anti-inflammatory, antioxidant, and anticancer effects^22–27^. Pit has been demonstrated exhibiting antibacterial and anti-biofilms effects to gram-negative bacteria based on multiple mechanisms^28–30^. In contrast, in this study, we found that pit and pts reduced the MIC values of ampicillin (Amp) against the *S. aureus* USA300 and improved the bactericidal capacity of Amp to *S. aureus* USA300 by targeting β-lactamase. Pit and ret didn’t exhibit antimicrobial properties, pts exhibited few antimicrobial abilities, but all these analogues inhibited the formation of *S. aureus* USA300 biofilm. Pit and its analogues bound to Hla directly and inhibited the hemolytic activity of the *S. aureus* USA300 strain culture supernatant. Pit reduced the cytotoxicity and the adherence of the *S. aureus* USA300 strain to human lung cancer epithelial cells, and protected *Galleria mellonella* (*G. mellonella*) from this pathogen infection when it was combined with or without Amp. Different from previous reports, this work finds that pit and its analogues exhibit antivirulent and anti-β-lactamase abilities simultaneously, promising their potential to be developed as adjuncts to treat *S. aureus* infections.

## Materials and methods

### Strains and reagents

The *S. aureus* USA300 strain that stored in our laboratory was originally purchased from the American Type Culture Collection. The compounds were purchased from Sichuan Ruifengshi Biotechnology Co., Ltd. *G. mellonella* was purchased from Tianjin Huiyude Biotechnology Co., Ltd. Cefnitrothiophene was obtained from Shanghai Yuanye Bio-Technology Co., Ltd.

### Protein preparation

The β-lactamase protein expression vector was constructed via a previously described method^31^, and the purified protein of β-lactamase and its mutants was obtained via a previously reported protocol^32^. Hla was stored in our laboratory and the Hla mutants were obtained via the same method. The primers used for this study are shown in Table 1.

**Table 1:** The primers used for this work.

Gene name	Primer name	Oligonucleotide (5’-3’)	Length (bp)
*hly*	R226A-F	CTTTTCATGAAAACTGCGAATGGTTCTATGAAAG	879
	R226A-R	CTTTCATAGAACCATTCGCAGTTTTCATGAAAAG	
	W205A-F	GTGAATCAAAATGCGGGACCATATG	
	W205A-R	CATATGGTCCCGCATTTTGATTCAC	
	M223A-F	GCAATCAACTTTTCGCGAAAACTAGAAATG	
	M223A-R	CATTTCTAGTTTTCGCGAAAAGTTGATTGC	
	Q267A-F	GAAAAGCATCCAAAGCGCAAACAAATATAG	
	Q267A-R	CTATATTTGTTTGCGCTTTGGATGCTTTTC	
	W312A-F	GAAAGATATAAAATCGATGCGGAAAAAGAAGAAATGAC	
	W312A-R	GTCATTTCTTCTTTTTCCGCATCGATTTTATATCTTTC	
	D261A-F	CAGTTATTACTATGGCGAGAAAAGCATCCAAAC	
	D261A-R	GTTTGGATGCTTTTCTCGCCATAGTAATAACTG	
*blaZ*	Y96A-F	GATATAGTTGCTGCGTCTCCTATTTTAG	771
	Y96A-R	CTAAAATAGGAGACGCAGCAACTATATC	
	I158A-F	GTTAGATATGAGGCGGAATTAAATTAC	
	I158A-R	GTAATTTAATTCCGCCTCATATCTAAC	
	K66A-F	CTTCAACTTCAGCGGCGATAAATAG	
	K66A-R	CTATTTATCGCCGCTGAAGTTGAAG	
	β-lac-F	CTGGGATCCGCCAAAGAGTTAAATGATTTA	
	β-lac-R	CTGGTCGACTTAAAATTCCTTCATTAC	

The underlines represent the mutation sites or the cleavage site, β-lac represents β-lactamase, F and R represent forwards and reverse respectively.

### Cefnitrothiophene hydrolysis assays

The inhibition experiments were carried out via a previously described method with some modifications^33^. Four micrograms of purified protein were incubated with different concentrations (0, 32, 64, or 128 µg/mL) of pit or its derivatives at 37 °C for 20 min, and then, cefnitrothiophene (5 µg) was added for an additional 10 min of incubation. The absorbance at 492 nm was measured to analyse the inhibitory effect of each compound on β-lactamase activity.

### Molecular docking and molecular modelling

The complex of *S. aureus* β-lactamase with its ligand was obtained from the RCSB Protein Data Bank (RCSB PDB; PDB ID of 6wgr). The structure of this complex was obtained via X-ray with a resolution of 1.88 angstroms (Å). The binding sites between the complex were predicted with the Protein-Ligand Interaction Profiler^34^. To explore the binding of the tested compounds to the β-lactamase, the protein was set as the receptor, and the analogues were used as ligands to perform docking calculations via AutoDock Vina^35^. Hydrogen atoms and charges were added to the proteins and ligands via AutoDockTools-1.5.6. The size of the docking box was 40 × 40 × 40 Å, with a spacing of 1 Å. Docking between Hla (PDB ID: 7ahl) and these compounds was performed via the same method. The binding modes of pit and Hla or β-lactamase were used to carry out molecular modelling with reference to methods reported previously to elucidate their interactive mechanism^36,37^.

### Minimum inhibitory concentration assay

The minimum inhibitory concentrations (MICs) of pit and its derivatives alone or in combination with antibiotics were determined according to the Clinical and Laboratory Standards Institute (CLSI). Briefly, Luria-Bertani (LB, hopebio, China) medium containing a series of different concentrations of Amp (0–128 µg/mL) were obtained by making double dilutions, pit or its derivatives were added (pit and ret 32 µg/mL, pts 32, 16, 8 µg/mL), and finally bacteria were added to reach a final concentration of 5 × 10^5^ colony-forming units per millilitre (CFU/mL). After cultured at 37 ℃ for 24 h, the lowest concentration that without visible bacterial growth was defined as the MIC. The synergies between gentamicin (Gm, 0–16 µg/mL) and pit derivatives (pit and ret 32 µg/mL, pts 8 µg/mL) were determined via the same method.

### Time-dependent bacterial killing

The *S. aureus* USA300 strain (approximately 5 × 10^5^ CFU/mL) was cultured in LB medium at 37 °C with shaking after treating with pit analogues (32 µg/mL), Amp (64 µg/mL) or their combination. Then, samples from different treatment groups were collected every two hours and plated on LB agar medium (10 µL) after dilution. Colonies were obtained after culture overnight at 37 °C to evaluate the effect of pit analogues on the bactericidal abilities of Amp.

### Biofilm assays

*S. aureus* (5 × 10^7^ CFU/mL) was cocultured with various concentrations of pit or its derivatives (0, 4, 8, 16 µg/mL for pts; 0, 4, 8, 16, 32 µg/mL for pit; 0, 8, 16, 32, 64 µg/mL for ret) at 37 °C for 24 h. The samples were washed three times with sterile phosphate-buffered saline (PBS) buffer after the culture medium was discarded. Then, 0.1% crystal violet was added to each well, and the samples were stained for 30 min. Next, glacial acetic acid (33%) was used to dissolve the stain after the samples were washed, and the absorbance of each sample was measured at 570 nm by using a microplate reader (BioTek, Synergy H1M).

### Growth curve and hemolytic activity assay

Different concentrations of the analogues (0, 8, 16 µg/mL for pts; 0, 16, 32 µg/mL for pit and ret) were cocultured with *S. aureus*, and the optical density (OD) at 600 nm of each sample was measured to evaluate whether the compound affects the growth of bacteria every one hour. The supernatant of each sample was obtained after centrifugation (12000 rpm, 5 min) and filtered with a 0.22-micron filter (BIOFIL, China); 50 µL of the supernatant was added to 450 µL sterile PBS that contained 12.5 µL of sterile defibrillated sheep blood and incubated at 37℃ for 10 min. Then, an equal volume of sample was used to detect the OD_543_ after centrifugation to measure the effects of these compounds on the hemolytic activity of the cultural supernatant.

### Cytotoxicity and adhesion detection

Human lung cancer epithelial A549 cells cultured in Dulbecco’s modified Eagle’s medium (DMEM, HyClone) supplemented with 10% foetal bovine serum (FBS, HyClone) were seeded in 96-well plates and cultured at 37 °C with 5% CO_2_. The next day, the cells were treated with *S. aureus* USA300 (multiplicity of infection: MOI = 50) with or without pit (32 µg/mL), Amp (64 µg/mL) or their combination for 6 h. Based on the metabolic dynamic parameters of pit, the MIC values of pit against *S. aureus* USA300 strains determined in this study, as well as the previous research foundation^38,39^, we selected 32 µg/mL of pit to perform exploration. The supernatant was used to detect lactate dehydrogenase (LDH) activity via a LDH detection kit (Beyotime Biotechnology, Shanghai, China) after centrifugation (1000 rpm, 10 min). The cells were treated with live/dead reagents (Beyotime Biotechnology, Shanghai, China) and incubated for 30 min in the dark after being washed with PBS. Images were obtained via a fluorescence microscope (OLYMPUS, IX83). Cells treated with 0.1% Triton X-100 or DMEM alone were used as positive control (PC) or negative control (NC), respectively. For the adhesion assay, A549 cells in 24 well plate (1.5 × 10^5^ cells/well) received treatment of *S. aureus* USA300 and different concentrations of pit (0, 16, 32 µg/mL) for 1 h. Then the medium was removed, and the cells were washed with sterile PBS. Each sample (10 µL) was coated to LB agar medium after dilution and cultured at 37 ℃ overnight. The clones were obtained to analyze the effect of pit against *S. aureus* USA300 adhere to A549 cells.

### *G. mellonella* infection model

Each *G. mellonella* individual was injected with 10 µL (5 × 10^7^ CFU/mL) of the *S. aureus* USA 300 strain. Four groups were established: a pit (50 mg/kg) treatment group, an Amp treatment group (12.5 mg/kg), a combination treatment group and a control group that was injected with an equal volume of solvent, and nine *G. mellonella* were assigned to each group. *G. mellonella* survival was monitored at predetermined time points to analyse the protective effects of different treatments against *S. aureus* infection. The degree of pathological tissue damage was determined via haematoxylin‒eosin (HE) staining.

### Statistical analysis

The data are presented as the means and standard deviations (SDs) or standard errors of the means (SEMs). An unpaired *t test* was used to determine the significance of differences by using GraphPad Prism 9.5.0. A p value ≤ 0.05 was considered to indicate statistical significance.

## Results

### Pit and its analogues bind to β-lactamase and inhibit its activity

**Fig. 1:**
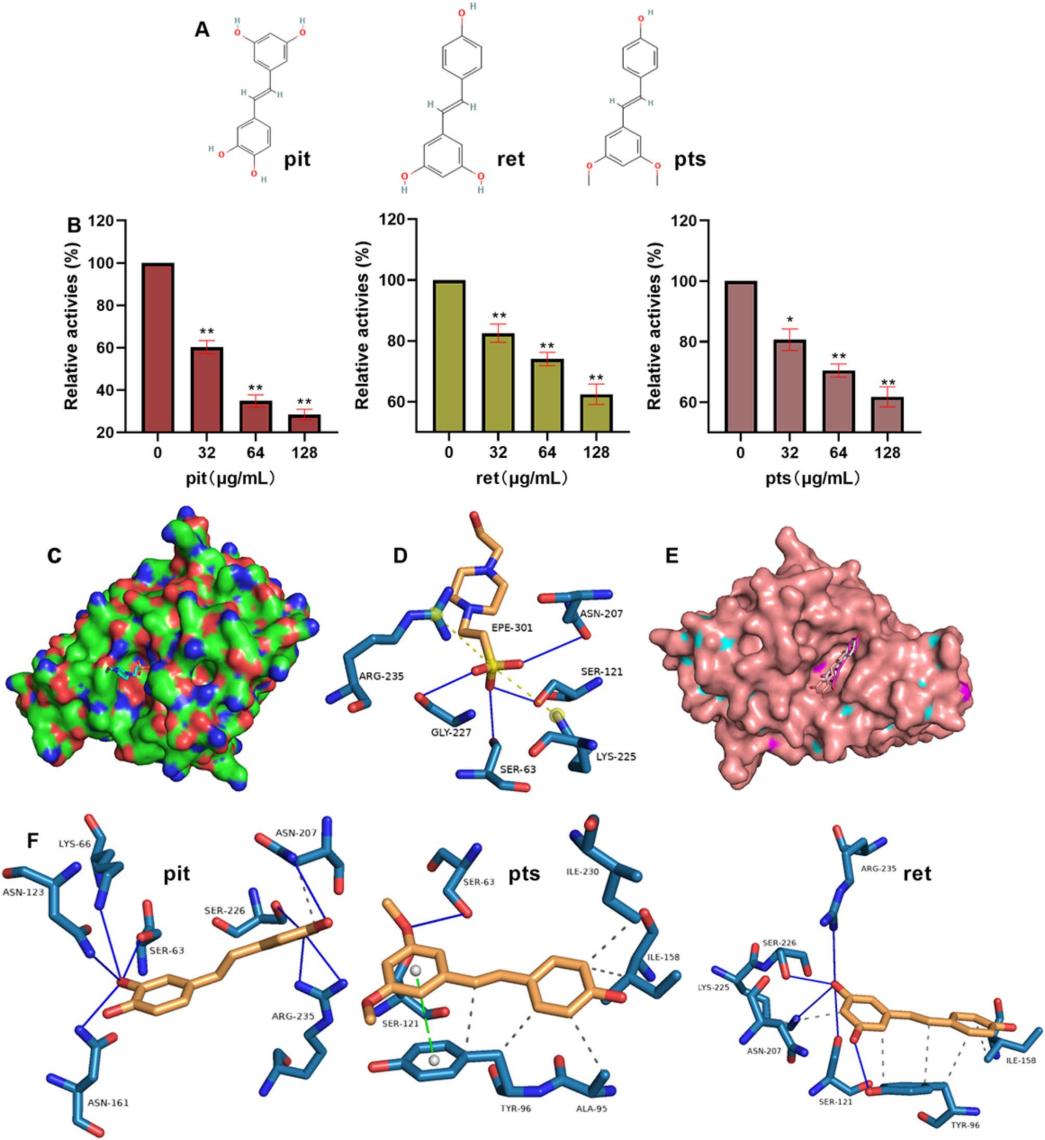
Pit analogues bind with β-lactamase and inhibit its activity. (**A**) Molecular structures of pit, ret and pts. (**B**) Inhibitory effects of pit, ret and pts on β-lactamase activity. Data are shown as the means with SEMs, *n* = 3, * indicates *p* ≤ 0.05, ** indicates *p* ≤ 0.01. (**C**) The complex of β-lactamase with its inhibitor and the binding sites (**D**). (**E**) The binding modes of pit analogues to β-lactamase and the binding sites between them (**F**).

β-lactamase hydrolyses β-lactam antibiotics to promote the resistance of *S. aureus.* Here, we found that pit and its analogues ret and pts (Fig. 1A) inhibited the hydrolysis of cefoperazone by β-lactamase, pit had the best inhibitory effect of 65.07% at 64 µg/mL, and inhibitory effects of 33.52% and 38.21% were detected when the concentrations of ret and pts were 128 µg/mL (Fig. 1B). These results indicate that these pit structural analogues can inhibit β-lactamase activity. In the structure of the complex of β-lactamase with its ligand EPE (Fig. 1C), hydrogen bonds formed between the β-lactamase residues SER63, SER121, ASN207, and GLY227 and EPE, and salt bridges formed between the β-lactamase residues LYS225 ARG235 and EPE (Fig. 1D), indicating that hydrogen bonds and salt bridges promote EPE binding. Pit, pts and ret bound to the same pocket of this protein (Fig. 1E), but the residues involved in the interactions were not exactly the same. For pit, hydrogen bonds were the critical binding forces, and the β-lactamase residues ASN123, LYS66, SER63, ASN161, SER226, ARG235 and ASN207 were involved in these interactions (Fig. 1F). Hydrophobic and π-stacking interactions were found between pts and the protein residues ALA95, TYR96, ILE158, ILE230, SER63, SER121 and TYR96 (Fig. 1F). Both hydrogen bonds and hydrophobic interactions formed when ret bound to the β-lactamase (Fig. 1F). These results suggest that although these analogues bind to the same pocket, they may have different mechanisms of action.

### Critical residues promoting pit binding with β-lactamase were identified

**Fig. 2:**
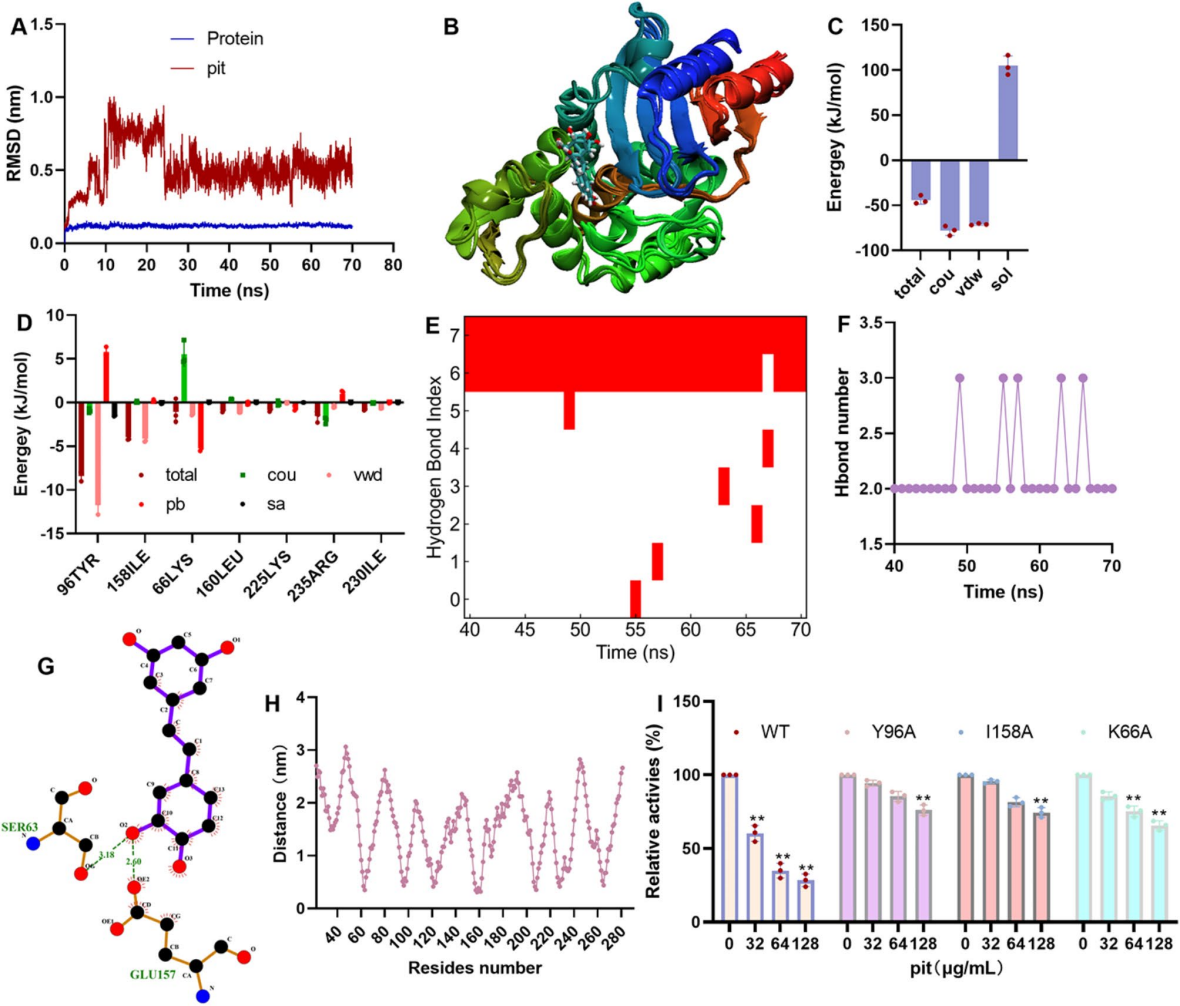
Interaction mechanism of pit and β-lactamase. (**A**) RMSD fluctuations of β-lactamase and pit during modelling. (**B**) The relative positions of proteins and ligand at different times. (**C**) Total binding free energy and residue energy decomposition (**D**) in the equilibrium stage. (**E**) Occupancy, number fluctuations (**F**) and details (**G**) of hydrogen bonds in the equilibrium stage. (**H**) The distance between residues and pit. (**I**) The inhibitory effect of pit on β-lactamase mutants. ** indicates *p* ≤ 0.01.

To explore the mechanism, the binding mode of pit and β-lactamase was used to perform a molecular modelling assay. The RMSD fluctuations of β-lactamase and pit were less than 0.1 nm after 30 ns (Fig. 2A). The configuration at different times revealed that the pit remained in the original binding pocket (Fig. 2B), confirming that the docking result was reliable. The total binding free energy (total) between them was − 44.02 ± 3.84 kJ/mol, which was composed of the Coulomb force (cou, -77.9 ± 4.35 kJ/mol), van der Waals force (vdw, -70.93 ± 0.83 kJ/mol) and solvation energy (sol, 104.80 ± 8.89 kJ/mol) (Fig. 2C). Residue energy decomposition revealed that 96TYR, 158ILE, 66LYS, 160LEU, 225LYS, 235ARG and 230ILE contributed more energy (Fig. 2D). Hydrogen bond analysis revealed that two pairs of H-bonds persisted (Fig. 2E and F) with occupancies of 96.8% and 100% (Fig. 2E). The donors were the oxygen atoms of the pit ortho-hydroxyl, and the OGs of SER63 and OE2 of GLU157 were the acceptors (Fig. 2G). These residues that interacted with pit showed a shorter distance than the others (Fig. 2H). Residue mutation was performed to confirm the critical residues, and the biological functions of the mutants were similar to those of the wild type (WT), but the inhibitory effects of pit on these mutants were significantly reduced compared with those on the WT (Fig. 2I), confirming that 158ILE, 96TYR and 66LYS were the core residues that promoted binding.

### Pit and pts enhance the bactericidal ability of Amp by increasing bacterial susceptibility

**Fig. 3:**
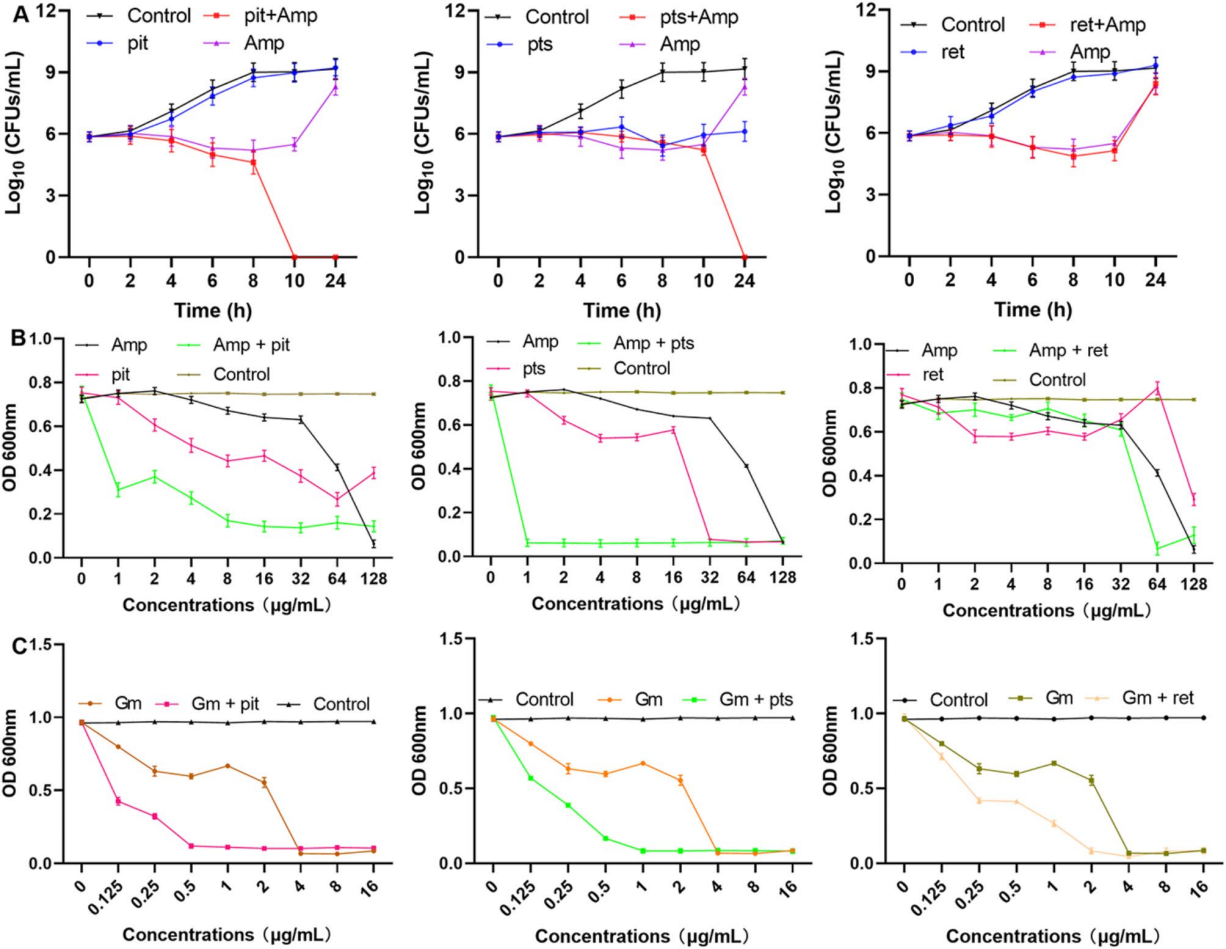
Pit analogues enhance the bactericidal ability of Amp by increasing bacterial susceptibility to antibiotics. (**A**) Logarithmic bacterial density value when the *S. aureus* USA300 were treated with Amp alone or in combination with pit, pts or ret. The data are shown as the means with SDs, *n* = 3. (**B**) OD_600_ values of *S. aureus* USA300 treated with Amp, Gm (**C**), pit, pts, ret or their combinations. The data are shown as the means with SEMs, *n* = 3.

The bacteria presented similar growth trends in the control or pit (32 µg/mL) treatment groups, almost no propagation was observed until 10 h of incubation in the Amp (64 µg/mL) group, proliferating bacteria were detected at 24 h. In the combination group, the number of clones decreased with time and could not be detected from 10 to 24 h (Fig. 3A), suggesting that pit addition improved the bactericidal ability of Amp. The bacterial density remained at its initial level when the bacteria were treated with 32 µg/mL pts. However, no clones were detected in the combined treatment group at 24 h, indicating that pts can also enhance the bactericidal capacity of Amp (Fig. 3A). Moreover, ret did not enhance the bactericidal ability of Amp, as the bacteria in this group presented the same growth trend as those in the isolated Amp treatment group did (Fig. 3A). MIC assays were carried out to evaluate whether these compounds affect the susceptibility of bacteria to antibiotics. The MICs values for pit, pts and ret were 64, 32, and 128 µg/mL (Table S1), and the MICs of Amp against *S. aureus* USA300 decreased from 128 µg/mL to 8 µg/mL or 1 µg/mL when 32 µg/mL pit or pts was coapplied (Fig. 3B). Regrettably, ret did not have a synergistic effect with Amp (Fig. 3B), in addition, the MICs of Amp against the *S. aureus* USA300 changed from 128 µg/mL to 8 µg/mL or 16 µg/mL when 16 µg/mL or 8 µg/mL pts was used (Fig. S1). Similarly, changes in the MICs were also observed when these compounds were coadministered with Gm (Fig. 3C). These results confirm that pit and pts increased the susceptibility of the *S. aureus* USA300 strain to Amp and Gm.

### Pit analogues inhibit the formation of *S. aureus* biofilm

**Fig. 4:**
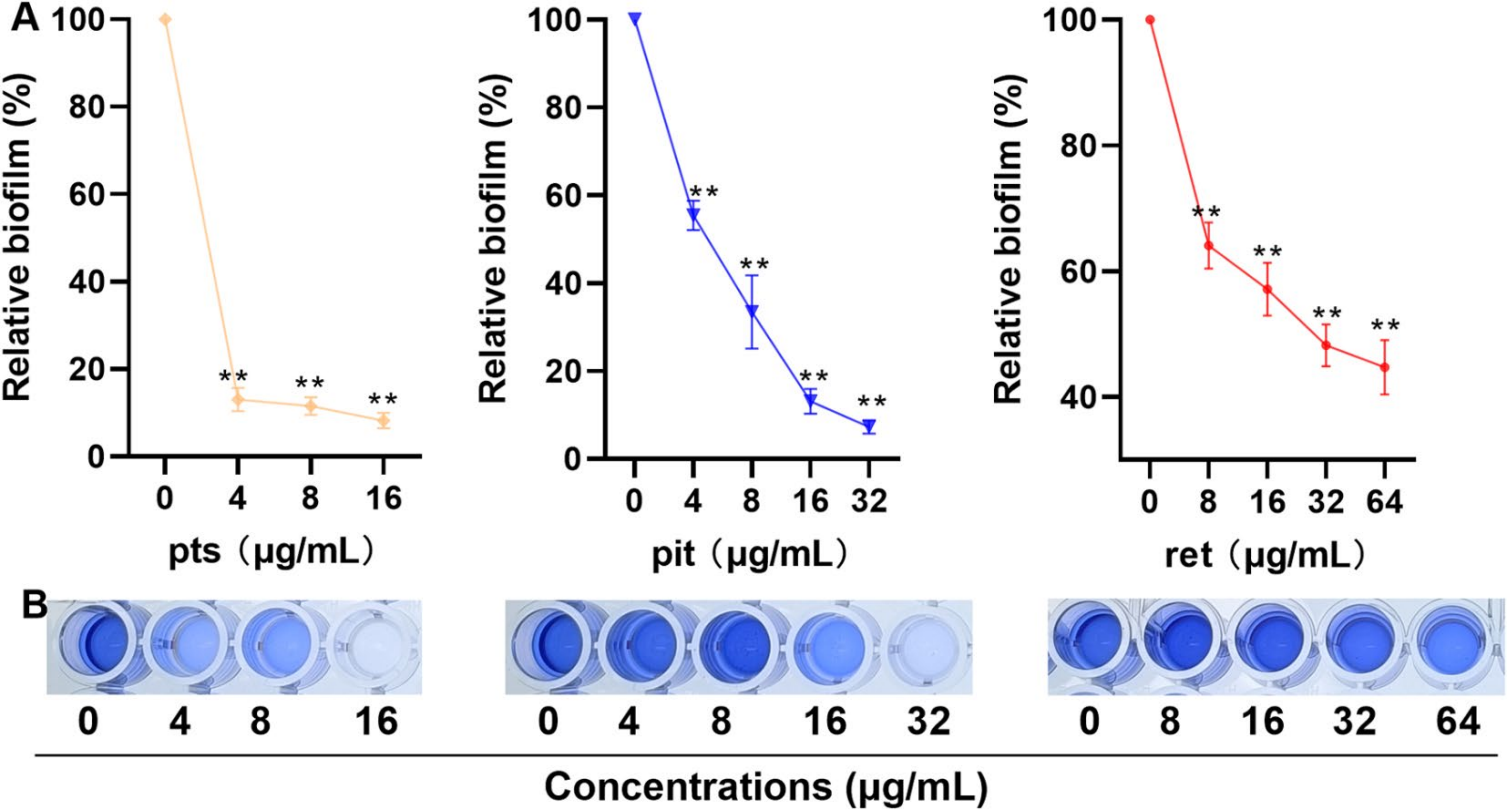
Pit analogues inhibited the formation of *S. aureus *biofilm. (**A**) The relative amounts of biofilms when the *S. aureus* USA300 were treated with appropriate concentrations of pts, pit or ret. The data are shown as the means with SDs, *n* = 3. ** indicates *p* ≤ 0.01. (**B**) Visual images of biofilms.

We analysed the effects of these analogues on *S. aureus* USA300 biofilm formation. A greater than 80% reduction in *S. aureus* USA300 biofilms was detected when the pts concentration was 4–16 µg/mL, for pit and ret, biofilm formation decreased gradually with increasing concentration. More precisely, pit and ret at 32 µg/mL reduced biofilm formation by approximately 90% and 50%, respectively (Fig. 4A), suggesting that these compounds reduce biofilm formation. The visual images of biofilm formation under different treatments are shown in Fig. 4B.

### Pit analogues bind with Hla to inhibit bacterial culture supernatant hemolysis activity

**Fig. 5:**
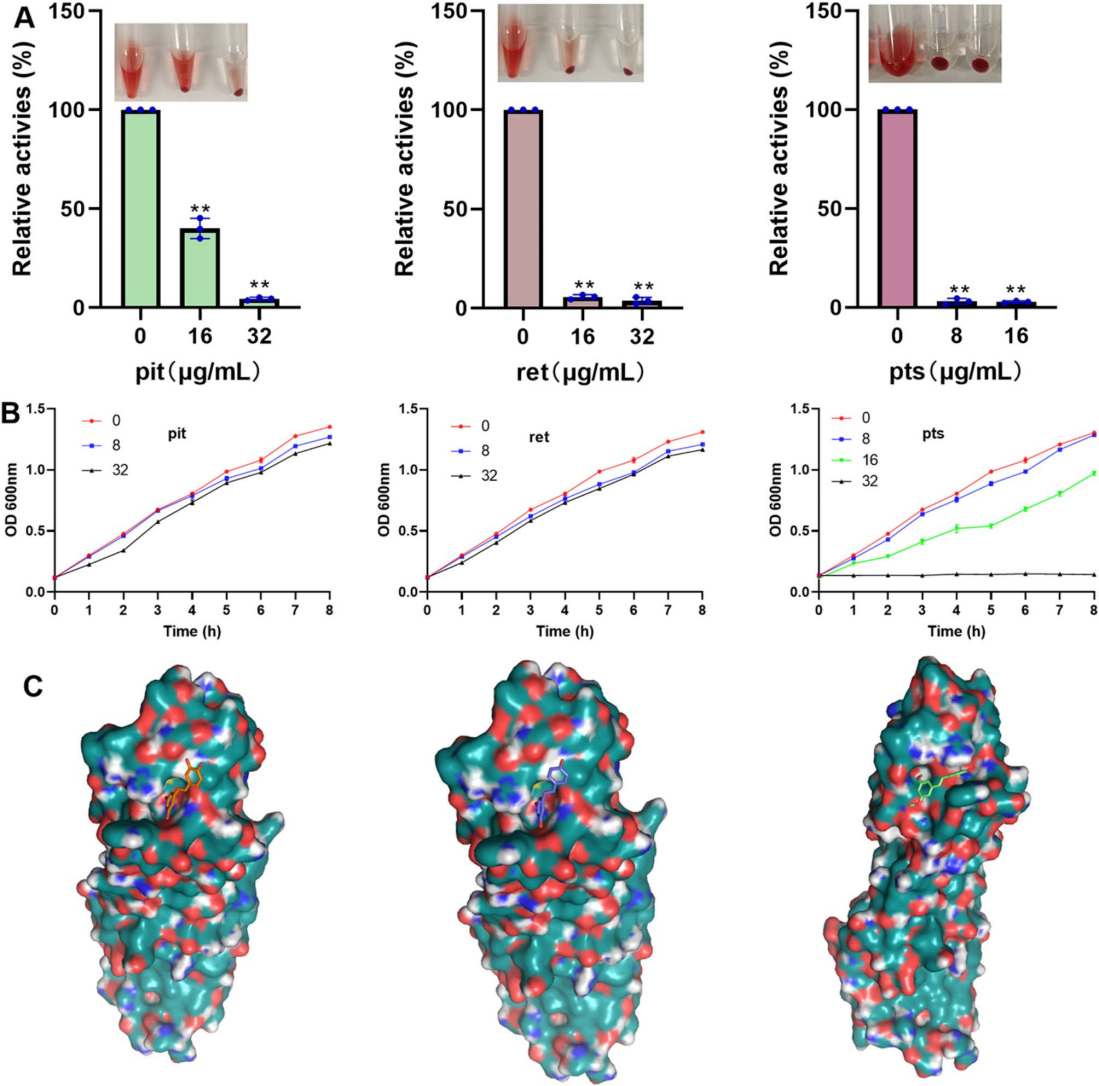
Pit analogues inhibited the hemolysis activity of the *S. aureus* USA300 culture supernatant by binding with Hla. (**A**) Inhibitory effects of the compounds on hemolysis activity. The data are shown as the means with SDs, *n* = 3. ** indicates *p* ≤ 0.01. (**B**) Effects of the analogues on the growth of the *S. aureus* USA300. (**C**) The binding mode between compounds and Hla.

The hemolysis activity of the bacterial culture supernatant decreased to 4.50% and 3.74% when the pathogen was cocultured with 32 µg/mL pit or ret, respectively, and 16 µg/mL pts reduced the hemolytic activity to 2.86% (Fig. 5A). Growth monitoring revealed that 32 µg/mL pit or ret did not affect the normal growth of bacteria, 16 µg/mL pts slowed bacterial growth, and 32 µg/mL pts inhibited the growth of bacteria (Fig. 5B). The molecular docking results revealed that the affinities of the three analogues for Hla were − 6.93 ± 0.05 kcal/mol, -6.33 ± 0.09 kcal/mol, and − 5.83 ± 0.05 kcal/mol for pit, ret, and pts, respectively, and these analogues bound to the same pocket of the protein (Fig. 5C). These results confirm that pit and ret do not affect growth at the test concentrations and inhibit the hemolysis activity of the supernatant by binding to Hla.

### Pit has a complicated interactive mechanism with Hla

**Fig. 6:**
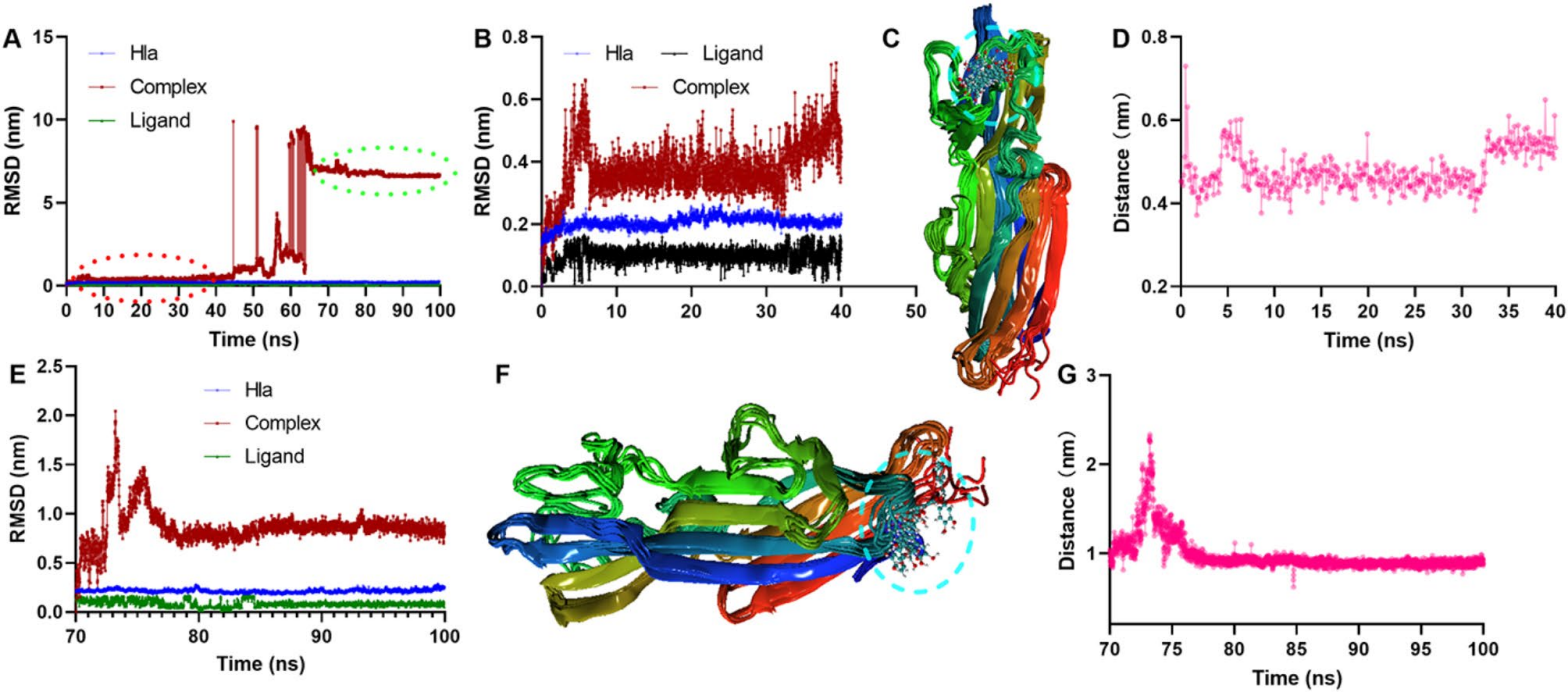
Binding mode between pit and Hla. (**A**) RMSD values during the 100 ns or 40 ns (**B**) of modelling. (**C**) The relative positions of Hla and pit at different times when it binds to the rim domain and the distance between pit and the reference residue (**D**). (**E**) RMSD values from 70–100 ns. (**F**) The binding pocket at the nitrogen terminal and configurations at different times. (**G**) The distance between pit and the arranged residue at 70–100 ns.

To explore the mechanism, the binding mode of pit and Hla was used for analysis. After a 100 ns simulation, we found that pit remained at the original rim domain binding pocket until 40 ns; then, it left and moved to the nitrogen terminal to find a pocket and stayed there (Fig. 6A). More precisely, the RMSD fluctuation (Fig. 6B), the relative positions at different times (Fig. 6C) and the distance between pit and 197MET (Fig. 6D) confirmed that pit remained in the original pocket. Similarly, for 70–100 ns, stable binding was evident by these indices and parameters (Fig. 6E‒G).

**Fig. 7:**
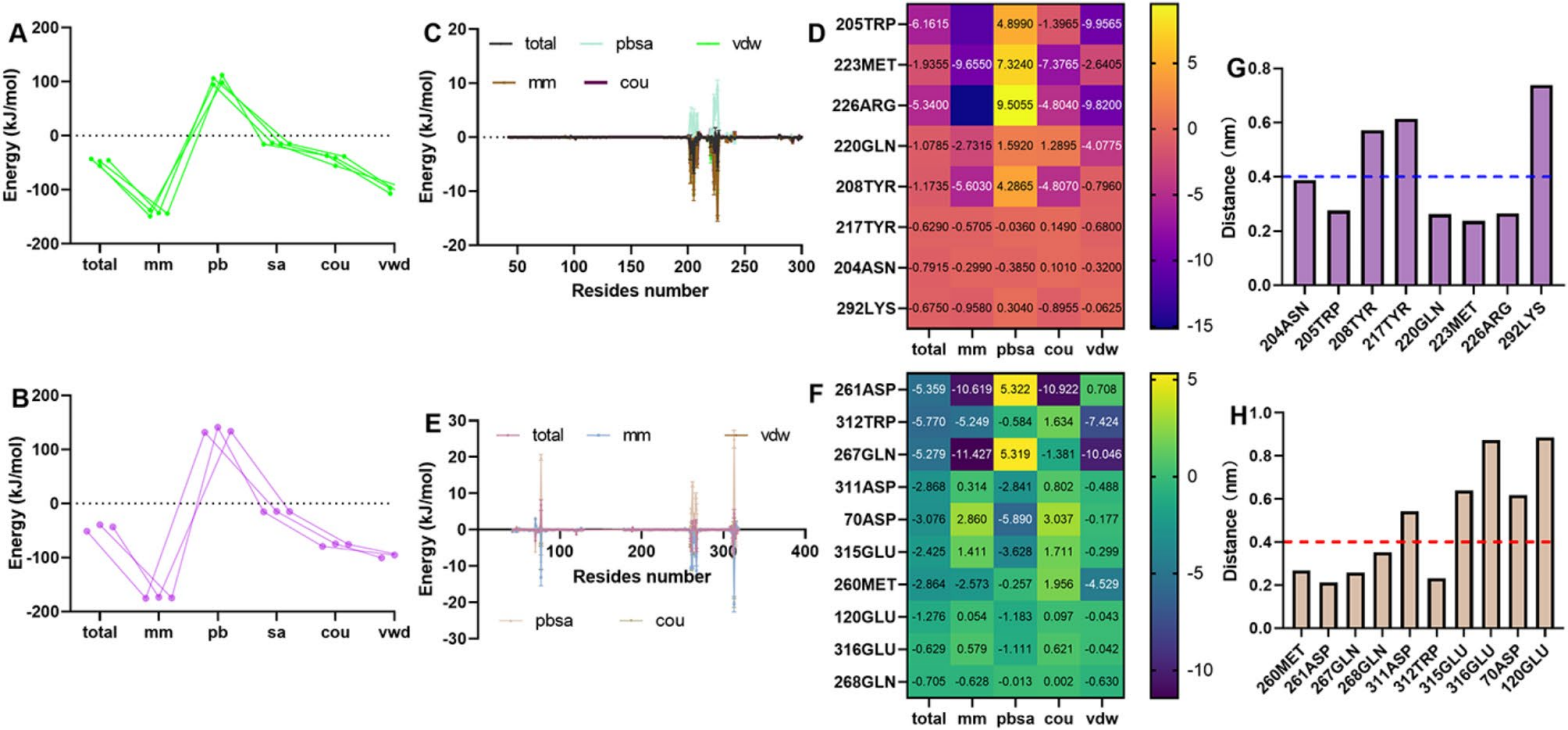
Analysis of residues that might interact with the Hla complex. (**A**) The total binding free energy when pit is located in a rim domain or nitrogen terminal (**B**) and the corresponding residue energy decomposition (**C**-**F**). (**G**) The distance between pit and residues for the rim domain binding mode or the nitrogen-terminal mode (**H**).

The energies of the two stable binding modes were analysed. For the original rim domain binding, the total binding free energy was − 47.81 ± 4.70 kJ/mol, including MM -143.20 ± 3.80 kJ/mol, which is composed of cou (-42.84 ± 7.46 kJ/mol) and vdw (-100.35 ± 5.59 kJ/mol), and the solvation energy was 88.06 ± 5.94 kJ/mol, which consists of polar (pb, 102.67 ± 6.63 kJ/mol) and nonpolar (sa, -14.61 ± 0.81 kJ/mol) interactions (Fig. 7A). Similarly, the energy values for the new nitrogen terminal binding mode were as follows: the total binding free energy was − 44.47 ± 5.01 kJ/mol, and the MM energy was − 174.39 ± 0.80 kJ/mol, which is composed of cou (-76.5 ± 2.03 kJ/mol) and vdw (-97.89 ± 2.20 kJ/mol). The solvation energy was 120.76 ± 3.77 kJ/mol, consisting of pb (135.62 ± 3.9 kJ/mol) and sa (-14.86 ± 0.18 kJ/mol) (Fig. 7B). Residue energy decomposition revealed that 205TRP, 223MET, 226ARG, 220GLN, etc., contributed more energy to rim domain binding (Fig. 7C and D), whereas 261ASP, 312TRP, 267GLN, 311ASP, etc., contributed more energy to the nitrogen terminal binding mode (Fig. 7E and F), and these residues were closer to pit (Fig. 7G and H).

**Fig. 8:**
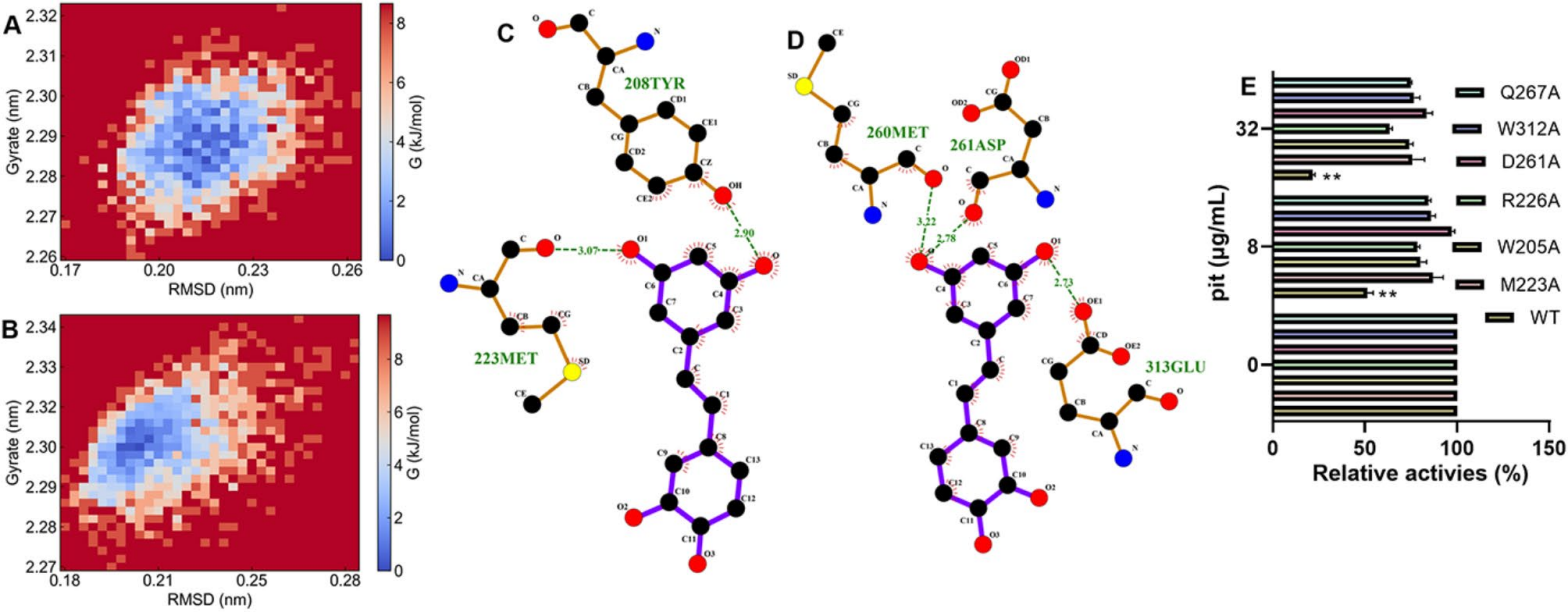
Confirmation of the critical residues that facilitate binding. (**A**) The binding free energy topography when pit bound at the rim domain or nitrogen terminal (**B**). (**C–D**) The details of the hydrogen bonds in the lowest energy conformation when pit bound at the rim domain or nitrogen terminal (**E**) Inhibitory effects of pit on WT Hla or its mutants. *n* = 3. ** indicates *p* ≤ 0.01.

The binding free energy topography analysis revealed that the two binding modes formed only one minimum energy pool (Fig. 8A and B). H-bonds existed in the minimum energy conformation. 208TYR and 223MET were involved in forming hydrogen bonds in the rim domain configuration (Fig. 8C). 260MET, 261ASP and 313GLU participated in the formation of hydrogen bonds in the nitrogen terminal binding mode (Fig. 8D), and the oxygen atoms of the intermediate hydroxyl of pit were involved in the hydrogen bonds (Fig. 8C and D**)**. 205TRP, 223MET and 226ARG were identified as the critical residues for rim domain binding, and 261ASP, 312TRP and 267GLN were confirmed to be important for promoting nitrogen terminal location, as pit almost lost its inhibitory effect on Hla after these residues were mutated (Fig. 8E).

### Pit reduces the cytotoxicity and the adherence of *S. aureus* USA300 to cells

**Fig. 9:**
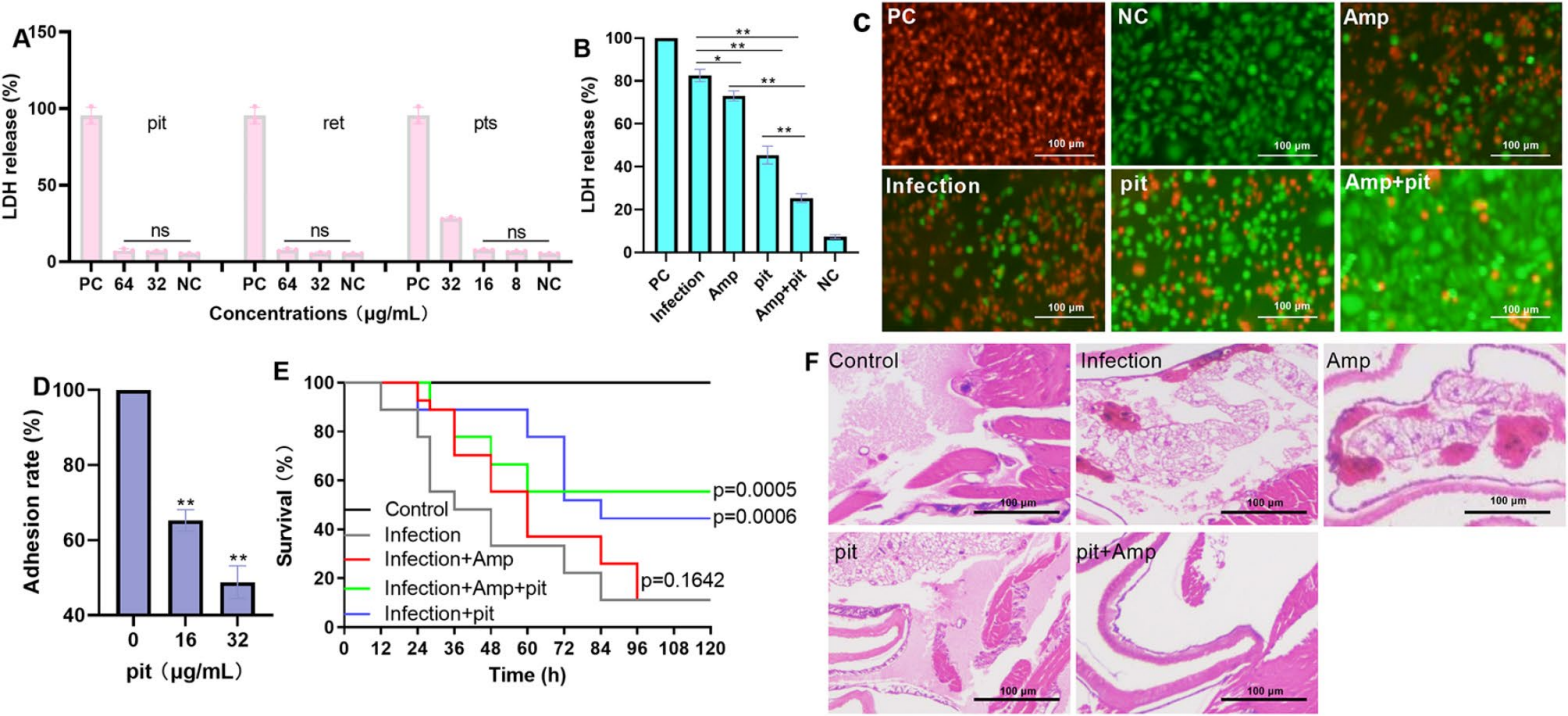
Pit reduces the cytotoxicity and protects *G. mellonella* from *S. aureus* infection. (**A**) The LDH levels of cells when treated with pit, ret or pts. The data are shown as the means with SEMs, ns means not significant. (**B**) The inhibitory effects of pit on cytotoxicity and the images of live and dead cell staining (**C**). PC represents positive control, NC represents negative control, infection represents cells treated with the *S. aureus* USA300 strain, pit or Amp represents cells that received *S. aureus* USA300 with pit or Amp treatment, respectively; and Amp + pit represents cells treated with *S. aureus* USA300, Amp and pit. The data are shown as the means with SEMs; ** indicates *p* ≤ 0.01. (**D**) Adhesion rate of the *S. aureus* USA300 to A549 cells treated with different concentrations of pit. The data are shown as the means with SEMs. ** indicates *p* ≤ 0.01. (**E**) Survival rates and degrees of injury (**F**) of *G. mellonella* in different treatment groups. The log-rank (Mantel‒Cox) method was used to analyse the survival rate of *G. mellonella*, and significance was defined as *p* ≤ 0.05; p values are shown in the graph.

The cytotoxicity of these analogues was measured by detecting LDH levels, and it was found that pit, ret and pts did not show cytotoxicity at 64–16 µg/mL, as the level of LDH from these samples was comparable to that of the negative control (Fig. 9A). Furthermore, the LDH level of cells from different treatments (Amp alone, pit alone or their combination) were much lower than that from the *S. aureus* USA300 alone treatment group (Fig. 9B), which was confirmed by the live/dead cell results (Fig. 9C). The inhibitory effect of pit on *S. aureus* USA300 adherence to A549 cells was explored, and 32 µg/mL pit reduced the adherence to 48.82% (Fig. 9D). Taken together, these findings suggest that pit reduced the cytotoxicity and adherence of *S. aureus* to cells, suggesting its potential for use in combating bacterial infection.

### Pit protects *G. mellonella* against *S. aureus* USA300 infection

Dead *G. mellonella* were detected at 12 h in the infection group, with a final survival rate of 11.11% (Fig. 9E). Deaths were detected in the Amp and pit treatment groups after 24 h of infection, and the survival rates of these groups were 11.11% and 44.44%, respectively (Fig. 9E). However, in the combination treatment group, the survival rate reached 55.56%, and the time to death was delayed (Fig. 9E), indicating that pit with or without Amp increased the survival rate and delayed the time to death. Distinct tissue destruction was observed in the infected and Amp treatment groups compared with the control group, but these tissue injuries were alleviated upon treatment with pit alone or in combination with Amp (Fig. 9F). These results provide evidence that pit can be used as an agent to treat infections caused by *S. aureus*.

## Discussion

Antibiotics play significant roles in ensuring the healthy growth of livestock and poultry, and antibiotics have been closely linked to the occurrence and spread of bacterial resistance^40^. The spread of antibiotic resistance complicates treatment^41^. Identifying methods to restore bacterial sensitivity to antibiotics, especially specific types of resistance, such as β-lactamase-mediated antibiotic resistance, can increase confidence in clinical treatment and alleviate the current challenges in antibiotic therapy.

In this study, pit and its analogues, which are mainly found in food ingredients, inhibited β-lactamase bioactivity via direct binding to the enzyme. However, different inhibitory effects were found for these compounds, although they share highly similar structures. Ret, which has a basic structure, led to an approximately 40% reduction in β-lactamase activity at 128 µg/mL, whereas pts, which has a methylated meta-hydroxyl group, did not have a notable increase in inhibitory effect. More than 70% inhibition was measured with pit, which has a hydroxyl group in contrast to the structure of ret. These data indicate that this hydroxyl group plays a key role in blocking the activity of the enzyme, which was confirmed by molecular docking, as this hydroxyl group formed four hydrogen bonds with four different residues of the β-lactamase. Mechanistic analysis showed that 96TYR, 158ILE and 66LYS were the key residues for pit and β-lactamase interaction, the identification of these residues could provide a basis for compound application or optimization.

Here, the synergistic effect of pit and its analogues with Amp and Gm was analyzed, it was found that the MICs values of Amp and Gm against *S. aureus* USA300 reduced at least 16 or 8 folds after treated with pit or pts, unfortunately, ret did not show obvious synergistic effect to Amp and Gm. Another pity is pts showed little antimicrobial properties, but pit did not affect the normal growth of *S. aureus* USA300, hence, pit possesses the potential to be used as an adjuvant of these two antibiotics to extend their sustainability of application.

Selective growth pressure and biofilm formation are important in promoting bacterial resistance^42,43^. *S. aureus* is the most common bacteria that forms biofilms upon infection, which can increase bacterial resistance, promote bacterial adhesion, and lead to long-lasting infection^10,44^. This study revealed that pit and its analogues significantly reduced the formation of *S. aureus* USA300 biofilms. Pit and pts showed better inhibitory effects than ret with more than 90% reduction, along with this, the adhesion of *S. aureus* USA300 strain to A549 cells was inhibited by pit with a dose dependent manner. Pit has been demonstrated inhibited biofilm formation of multiple gram-negative bacteria such as *Pseudomonas aeruginosa*,* Acinetobacter baumannii*, and *Klebsiella pneumoniae*^28^, the inhibitory effects of pit on *S. aureus* biofilm and adhesion could extend its application, but the underlying mechanisms need to be further explored. LDH levels from A549 cells treated with pit showed this compound didn’t have cytotoxicity to ensure the security for its application. Further, pit suppressed the cytotoxicity and protected *G. mellonella* from *S. aureus* USA300.

The Hla N-terminus plays an important role in the correct assembly of the heptamer, and the number of toxins assembled on the cell surface is reduced when the N-terminus is truncated, resulting in reduced cytotoxicity^45,46^. Membrane perforation cannot occur after mutation of some residues at the N-terminus^47^. The C-terminus is associated with membrane binding during the infection, the residues in the rim domain promote the binding of Hla to the phospholipid molecule of cell membrane^45,46^. In this study, pit initially bound to the rim domain of Hla, but in the simulation process, it moved to the N-terminus and remained there. The binding energies of the two binding modes were comparable. The reduction in the hemolysis activity of the culture supernatant confirmed this binding. The residues that promote the binding of pit to Hla at the rim domain involved 226ARG, 205TRP and 223MET, while residues of 267GLN, 312TRP and 26ASP in Hla engaged in the N-terminus interaction with pit. These results suggest that pit may bind to both the N-terminus and rim domain of Hla during the actual binding process to affect its biological function.

Overall, our work identifies promising compounds that have the potential to restore antibiotic efficacy and achieve excellent bactericidal effects with lower antibiotic doses or mitigate acute infection treatment failure upon combination treatment and slow the transition to chronic disease. These compounds can also play roles in the treatment of biofilm-related diseases and alleviate the current situation of severe drug resistance.

## Conclusion

Pit and its analogue pts increased the susceptibility of *S. aureus* to antibiotics by targeting β-lactamase and enhancing the bactericidal ability of Amp. These compounds inhibit the formation of *S. aureus* biofilms; they bind with Hla at both the rim domain and N-terminus to suppress hemolytic activity. Pit reduced the cytotoxicity and adherence of *S. aureus* to cells and protected *G. mellonella* from *S. aureus* infection. The identification of key residues involved in the interaction of pit with β-lactamase and Hla provides a theoretical basis for its optimization and modification. These results identify promising compounds for the development of *S. aureus* infection inhibitors.

## Electronic supplementary material

Below is the link to the electronic supplementary material.

Supplementary Material 1

## Data Availability

Data is provided within the manuscript or supplementary information files.
