# Supplementary material for: Piceatannol and its analogues alleviate Staphylococcus aureus pathogenesis by targeting β-lactamase biofilms and α-hemolysin
Source: Sci Rep. 2025 Feb 14;15:5551. doi: 10.1038/s41598-025-89654-1 (PMC11828952; doi:10.1038/s41598-025-89654-1)
Supplement: Supplementary file 1 — Supplementary Material 1 [file 41598_2025_89654_MOESM1_ESM.pdf]

Supplementary data for

Piceatannol and its analogues alleviate *Staphylococcus aureus* pathogenesis by targeting β-lactamase biofilms and α-hemolysin

Guizhen Wang[1], Jingyao Wen[1], Zizeng Tian[1], Hanbing Zhou[1], Xinli Peng[1], Peigang Zhang[1], Zhandong Li[1*]

[1]College of Biological and Food Engineering, Jilin Engineering Normal University, Changchun, 130052, China

***Correspondence:** Zhandong Li, Jilin Engineering Normal University, Changchun, 130052, China. Email:lizd591@jlenu.edu.cn

Table S1 The MIC values of pit, ret and pts against the *S. aureus* USA300 strain

**Compound**	**MIC** (μg/mL)
pit	64
ret	128
pts	32

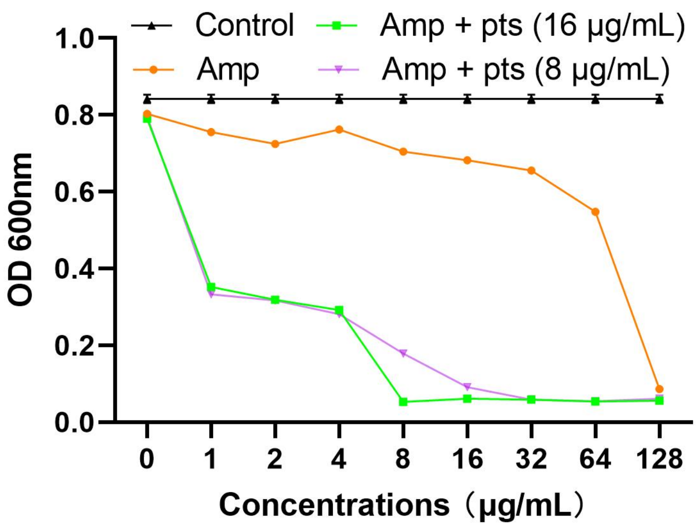

Figure S1 The changes of MIC values of Amp against the *S. aureus* USA300 strain when the bacteria were treated with Amp and different concentrations of pts.
